# Dual GIP/GLP1-RA, GCGR/GLP-1 RA and GLP1-RA for the Treatment of Metabolic Dysfunction-associated Steatotic Liver Disease with Type 2 Diabetes: A Systematic Review and Meta-analysis

**DOI:** 10.17925/EE.2025.21.2.5

**Published:** 2025-10-23

**Authors:** Burhan Gunawan, Heri Nugroho, Roy Panusuan Sibarani

**Affiliations:** 1. Diabetes Initiative Indonesia, Sumber Waras Hospital, West Jakarta, Indonesia; 2. Department of Internal Medicine, Faculty of Medicine, Diponegoro University, Central Java, Indonesia; 3. EMC Sentul Hospital, Bogor, Indonesia

**Keywords:** Glucagon-like peptide 1, glucagon receptor, glucose-dependent insulinotropic peptide, liver steatosis, type 2 diabetes

## Abstract

Previous studies have revealed that glucagon-like peptide-1 receptor agonist (GLP-1RA) can improve metabolic dysfunction-associated steatotic liver disease (MASLD) in individuals with type 2 diabetes (T2D). However, comprehensive research comparing dual glucose-dependent insulinotropic polypeptide (GIP)/GLP-1RA, glucagon receptor (GCGR) agonist/GLP-1RA and GLP-1RA is limited. This meta-analysis aimed to summarize the current evidence for the efficacy and safety of dual GLP/GIP-1RA, GCGR/GLP-1RA and GIP-1RA for these individuals. PubMed, Web of Science, Scopus and the Cochrane database were searched for randomized controlled trials that explore the efficacy of dual GIP/GLP-1RA, GCGR/GLP-1RA or GLP-1Ras for MASLD and T2D. The outcomes were the reversal of liver fibrosis degree and liver fat content (LFC) calculated using magnetic resonance imaging scan. The random-effects model was used to calculate the mean difference (MD) and odds ratio (OR) with a 95% confidence interval (CI). Thirteen studies with a total pooled sample of 1,552 individuals were included in the study. Dual GIP/GLP-1RA, GCGR/GLP-1RA and GLP-1RA were significantly superior in reversing the liver fibrosis degree (OR 3.72; 95% CI: 2.72, 5.09; p<0.001) and decreasing the LFC (MD -18.90; 95% CI: -18.43, -19.37; p<0.001) compared with other active therapies or placebo. Dual GIP/GLP-1RA (OR 28.90) and GCGR/GLP-1RA (OR 35.31) have greater efficacy in the reduction in LFC than single GLP-1RA (OR 8.23). Medications combining GIP/GLP-1RA and GCGR/GLP-1RA could be beneficial for individuals with both T2D and MASLD.

Fatty liver is the largest liver disease globally and has become a leading cause of liver cirrhosis and end-stage liver disease. Metabolic dysfunction-associated steatotic liver disease (MASLD), formerly known as non-alcoholic steatohepatitis (NASH), affects about one-third of the adult population worldwide, with its prevalence rising from 21.9% in 1991 to 37.3% in 2019.^1,2^ The highest prevalence rates have been observed in the Middle East and South America regions.^3^ MASLD is diagnosed based on histological, imaging or blood biomarker evidence of fat accumulation in the liver (hepatic steatosis), in addition to one of the following three criteria: overweight/obesity, presence of type 2 diabetes (T2D) or evidence of metabolic dysregulation.^1^ In individuals with T2D, the prevalence of MASLD reaches up to 65%, and 17% of individuals have advanced fibrosis.^4^ T2D and fatty liver have a strong bidirectional relationship. *De novo* hepatic lipogenesis and hyperglycaemia are the major pathways in fatty liver development. The excess level of circulating free fatty acids causes further worsening of insulin resistance.^5^

Recently, the connection between T2D and MASLD has gained significant attention, with a few pieces of evidence confirming that diabetes medication could improve MASLD and liver fibrosis.^6^ In a network meta-analysis by Ren et al., which evaluated 26 hypoglycaemic agents for MASLD, only empagliflozin (sodium-glucose co-transporter two inhibitor agent) and liraglutide (glucagon-like peptide-1 receptor agonist [GLP-1RA] agent) showed effectiveness in reducing liver stiffness, improving liver enzyme and improving insulin resistance. Meanwhile, pioglitazone showed limited benefits in improving metabolic dysfunction in this network meta-analysis.^7^

In recent years, glucagon-like peptide-1 (GLP-1) agonists have attracted considerable attention as medical therapy for T2D and MASLD. GLP-1 is a peptide hormone synthesized in L-cells located in the intestinal mucosa, alpha cells of the pancreatic islets and neurons in the nucleus of the solitary tract.^8^ The meta-analysis from Zhu et al. with eight trials and a total of 468 subjects showed that the administration of GLP-1 receptor agonists (GLP-1RAs) significantly decreased the content of intrahepatic adipose (p=0.007).^9^ Furthermore, several randomized controlled trials (RCTs) have shown good efficacy and safety of GLP-1 compared with placebo or insulin for MASLD, although the sample size was small.^10–12^ The GLP-1 receptor has been identified in hepatocytes, which could lower intrahepatocyte accumulation of fatty acid and promote oxidation of fatty acid.^13,14^

Unlike the GLP-1R, the liver is rich in glucagon receptors (GCGRs) and glucose-dependent insulinotropic polypeptide (GIP) receptors. Glucagon and GIP exert direct effects by stimulating hepatic beta-oxidation of fatty acids and reducing *de novo* lipogenesis. Tirzepatide (GIP/GLP-1 receptor agonist) and survodutide/pemvidutide (GPCR/GLP-1 receptor agonist weekly once) have been shown to induce significant weight reduction in individuals with T2D and obesity.^15–17^ Recent studies have demonstrated the beneficial metabolic effects of tirzepatide on the liver by alleviating oxidative stress and inflammation through modulation of the lipid metabolism pathway.^18^ The studies show that tirzepatide could reduce key inflammatory markers, such as YKL-40, intracellular adhesion molecule 1, C-reactive protein, leptin and growth differentiation.^19^

In a recent study involving individuals with T2D and MASLD, treatment with tirzepatide for 52 weeks resulted in greater reductions in liver fat and improvements in MASLD biomarkers compared with placebo.^20^ To date, no study has compared the effectiveness of GCGR/GLP-1RA and GIP/GLP-1RA for MASLD comprehensively. The evaluation of those studies requires meta-analysis. We hypothesized that improvement in intrahepatic lipid content would be significantly greater in dual GCGR/GLP-1RA, GLP/GIP-1RA and GLP-1RA compared with other therapies or placebo for MASLD. This systematic review and meta-analysis were performed to summarize current evidence for the efficacy and safety of dual GCGR/GLP-1RA, GLP/GIP-1RA and GLP-1RA in these individuals.

## Material and method

### Protocol

This research was conducted in accordance with the Preferred Reporting Items for Systematic Review and Meta-Analysis Guideline (PRISMA). The protocol for the review was registered with PROSPERO (Dual GIP/GLP1-RA and GLP1-RA for the Treatment of Metabolic dysfunction-associated Fatty Liver Disease [MAFLD] with Type 2 Diabetes Mellitus [T2DM]: A Systematic Review and Meta-Analysis; registration number: CRD42025642864).^21^

**Figure 1:**
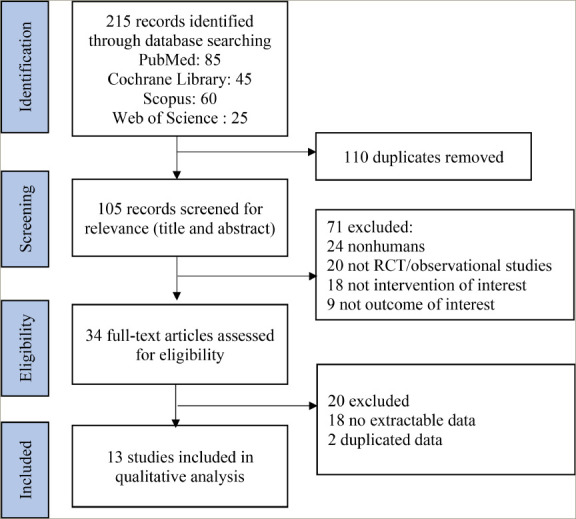
Article selection flow diagram

*RCT = randomized controlled trial*

**Table 1:** Study characteristics^11,12,20,22–31^

Number	Author (year)	Analysis	Subject	Age (mean ± SD); female sex (%)	Intervention (n)	Comparison (n)	Follow-up period	Method for liver fibrosis analysis	Fibrosis regression in the intervention group (n, %)	Fibrosis regression in the comparison group (n, %)	Percentage LFC reduction in the intervention group (mean ± SD)	Percentage LFC reduction in the comparison group (mean ± SD)
1	Loomba et al. (2024)^20^	Per protocol	MASH-confirmed, stage F2–F3	54.4 ± 11.3; 109 (57.0%)	Tirzepatide 5 mg (47), 10 mg (47), 15 mg (48)	Placebo (48)	52 weeks	Liver biopsy	5 mg: 26 (55%); 10 mg: 24 (51%); 15 mg: 24 (51%)	14 (30%)	5 mg: 45.7 ± 8.0; 10 mg: 41.3 ± 7.7; 15 mg: 57.0 ± 8.1	9.8 ± 8.2
2	Gastadelly et al. (2022)^22^	ITT	T2D with fatty liver index at least 60	56 ± 10; 124 (41.9%)	Tirzepatide 5 mg (71), 10 mg (79), 15 mg (42)	Titrated degludec (74)	52 weeks	Liver biopsy	5 mg: 47 (66.9%); 10 mg: 64 (81.4%); 15 mg: 33 (78.8%)	24 (32.1%)	5 mg: 29.8 ± 5.9; 10 mg: 47.1 ± 7.4; 15 mg: 39.6 ± 6.3	11.2 ± 2.3
3	Dutour et al. (2016)^11^	ITT	T2D and Obese	52 ± 1; 23 (52.3%)	Exenatide 10 µg bid (22)	Oral medication (22)	26 weeks		(-)	(-)	23.8 ± 9.5	12.5 ± 9.6
4	Armstrong et al. (2016)^12^	ITT	T2D and biopsy-confirmed NASH	51 ± 11; 14 (31.1%)	Liraglutide 1.8 mg/d (23)	Placebo (22)	48 weeks	Liver biopsy	9 (39%)	2 (9%)	(-)	(-)
5	Sanyal et al. (2024)^23^	ITT	T2D, biopsy confirmed MASH and fibrosis	50.8 ± 12.8; 155 (53%)	Survodutide 2.4 mg (73), 4.8 mg (72), 6 mg (74) once weekly	Placebo (74)	48 weeks	Liver biopsy	2.4 mg: 34 (74%); 4.8 mg: 47 (62%); 6 mg: 32 (43%)	10(14%)	2.4 mg: 50.9 ± 8.8; 4.8 mg: 62.8 ± 10.4; 6 mg: 64.3 ± 9.7	7.3 ± 1.5
6	Feng et al. (2017)^25^	ITT	T2D and IHF >10%	47.15 ± 1.17; 27 (29.0%)	Liraglutide 0.6 mg/day (30)	Metformin 500 mg3x/day (31); gliclazide 30 mg/day (32)	24 weeks		(-)	(-)	23.6±1.8	Metformin: 16.7 ± 2.2; gliclazide: 13.4 ± 1.4
7	Jinhua et al. (2019)^26^	ITT	T2D and IHF >10%	43.1 ± 9.7; 23 (30.7%)	Liraglutide 1.8 mg/day (24)	Sitagliptin 100 mg/d (27);glargine (24)	26 weeks		(-)	(-)	4.0 ± 4.5	Sitagliptin: 3.8 ± 5.0; glargine: 0.8 ± 5.3
8	Liu et al. (2020)^27^	Per protocol	T2D and IHF >10%	47.63 ± 10.14; 33 (43.4%)	Exenatide 5 pg 2x/day (38)	Glargine (38)	24 weeks		(-)	(-)	17.6 ± 12.9	10.5 ± 11.4
9	Kuchay et al. (2020)^28^	Per protocol	T2D and IHF >6%	48.1 ± 8.9; 19 (29.7%)	Dulaglutide 1.5 mg/week (32)	Standard treatment (32)	24 weeks		(-)	(-)	32.1 ± 4.3	5.7 ± 7.9
10	Newsome et al. (2020)^29^	ITT	T2D, Biopsy confirmed NASH	55.2 ± 10.9; 194 (84.3%)	Semaglutide 0.1 mg (57), 0.2 mg (59), 0.4 mg (56)	Placebo (58)	72 weeks	Liver biopsy	0.1 mg: 28; 0.2 mg: 19; 0.4 mg: 24	19	(-)	(-)
11	Bizino et al. (2019)^30^	ITT	T2D with NASH	60 ± 6; 20 (40.8%)	Liraglutide 1.8 mg 1x/day (23)	Placebo (26)	26 weeks		(-)	(-)	6.3 ± 7.1	4.0 ± 4.6
12	Sathyanarayana et al. (2012)^31^	Per protocol	T2D with NAFLD	52 ± 3; 10 (47.6%)	Exenatide 10 µg 2x/day (11)	Pioglitazone 45 mg/d (10)	52 weeks		(-)	(-)	11.0 ± 3.1	6.5 ± 1.9
13	Harrison et al. (2025)^24^	ITT	T2D with MASLD	47.9 ± 14; 50 (53.2%)	Pemvidutide weekly 1.2 mg (23), 1.8 mg (23), 2.4 mg (24)	Placebo (24)	12 weeks		(-)	(-)	1.2 mg: 8.9 ± 4.3; 1.8 mg: 14.7 ± 7.4; 2.4 mg: 11.3 ± 5.1	0.2 ± 2.1

*IHF = intrahepatic fat; ITT = intention to treat; LFC = liver fat content; MASH = Metabolic dysfunction-associated steatohepatitis; MASLD = metabolic dysfunction-associated steatotic liver disease; NAFLD = non-alcoholic fatty liver disease; NASH = non-alcoholic steatohepatitis; SD = standard deviation; T2D = type 2 diabetes.*

**Figure 2:**
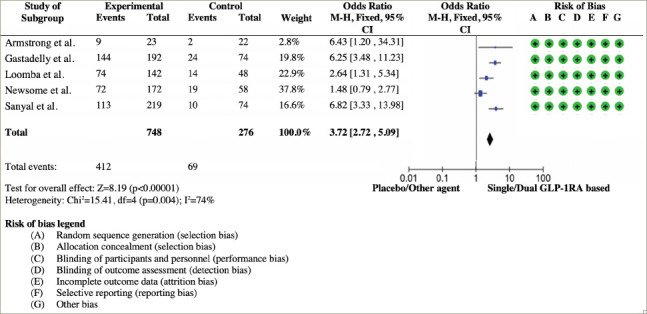
Forest plot analysis and risk of bias from studies with outcome reversal of liver fibrosis degree^1,12,20,22,23,29,32,33^

*CI = confidence interval; GLP-1RA = glucagon-like peptide-1 receptor agonist*

### Search strategy

We conducted a comprehensive search to find relevant studies from MEDLINE (PubMed), Science Direct, Scopus and the Cochrane Central Register for controlled trials published up to 20 January 2025, using the following combination of MeSH key terms: dual GIP/GLP-1RA, GCGR/GLP-1RA, GLP-1RA, tirzepatide, survodutide, pemvidutide, liraglutide, exenatide, semaglutide, dulaglutide, T2DM, diabetes mellitus type 2, metabolic dysfunction-associated steatotic liver disease (MASLD), metabolic dysfunction-associated fatty liver disease (MAFLD), non-alcoholic fatty liver disease, NAFLD, non-alcoholic steatohepatitis, NASH and RCT. Language restrictions were not applied.

### Study selection

Using a PICO framework, we selected studies for review that included the following: (1) an adult population with a definitive diagnosis of T2D and MASLD/MAFLD/NAFLD/NASH; (2) those who received dual GIP/GLP-1Ras, GCGR/GLP-1RA or GLP-1RAs for at least 24 weeks; (3) studies using placebo or other active treatments for comparison and (4) studies that provided data on liver fibrosis degree and its reversal assessment only by liver biopsy and liver fat content (LFC) by magnetic resonance imaging (MRI) scan. We excluded non-randomized, observational studies, reviews, editorials and studies involving secondary liver disease. The two reviewers (BG and HS) independently screened the literature and resolved any conflicts through discussion.

**Figure 3:**
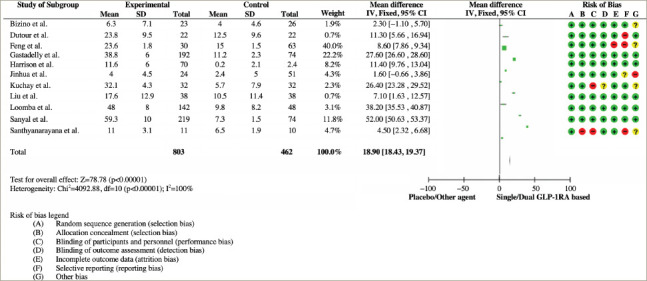
Forest plot analysis and risk of bias from studies reporting a reduction in liver fat content^11,19,20,22–28,30–33^

*CI = confidence interval; GLP-1RA = glucagon-like peptide-1 receptor agonist; SD = standard deviation*

**Figure 4:**
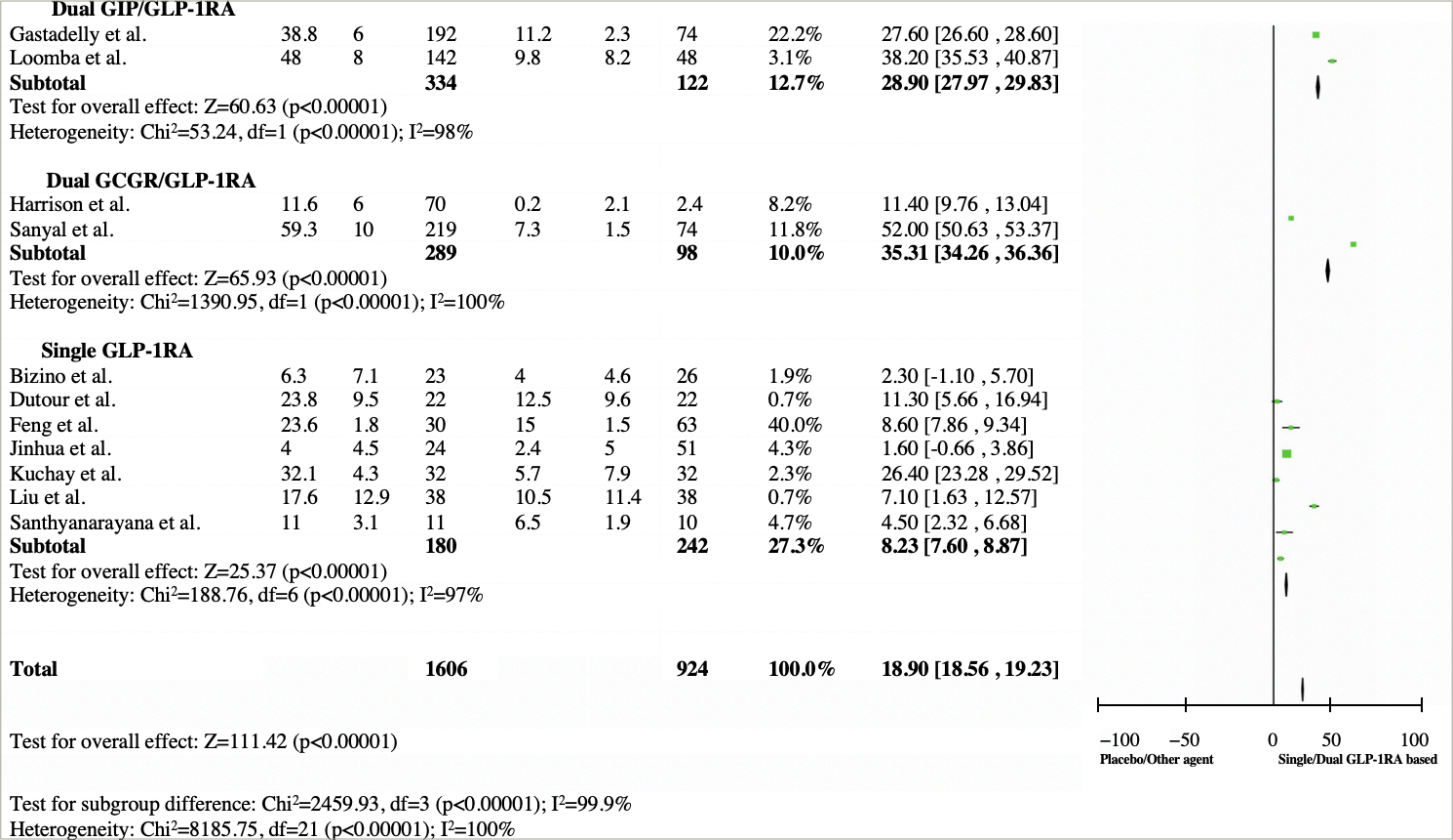
Forest plot analysis and risk of bias from subgroup studies^11,19,20,22–28,30–33^

*GCGR = glucagon receptor; GIP = glucose-dependent insulinotropic polypeptide; GLP-1RA = glucagon-like peptide-1 receptor agonist*

### Data extraction and risk-of-bias appraisal

Two reviewers (BG and HS) independently collected data from the selected studies, including author details, publication year, patient demographics, blinding method, study arms, sample size, treatment specifics and primary outcome assessment. We also extracted data on liver fibrosis degree reversal (minimum of one stage) and LFC from MRI scans. The quality of each RCT was then independently assessed by the same reviewers using the Cochrane Collaboration Tool, which evaluates seven domains: random sequence generation (selection bias), allocation concealment (selection bias), blinding of participants and personnel (performance bias), blinding of outcome assessment (detection bias), incomplete outcome data (attrition bias), selective reporting (reporting bias) and other bias. Discrepancies between reviewers were resolved through discussion. The process of data extraction is detailed in *Figure 1*.

### Statistical analysis

We analysed both dichotomous and continuous variables. Dichotomous data were presented as numbers (percentages) and continuous data were presented as means ± standard deviations (SDs). To synthesize continuous data, we calculated the weighted mean difference with a 95% confidence interval (CI). The *I*^2^ statistic was used to determine study heterogeneity. We used a random-effects model to pool continuous variables when *I*^2^>50% (considered as high heterogeneity). We also performed the subgroup analysis to compare the LFC reduction between dual GCGR/GLP-1RA, dual GLP/GIP-1RA and single GLP-1RA individually. All statistical calculations were performed using RevMan version 5.4 software (Cochrane Collaboration, Oxford, UK).

## Results

### Baseline characteristics

Initially, we identified 215 records from four databases; finally, only 13 studies were included in the final meta-analysis. The large exclusion of the records identified was because we analysed only those studies that used liver biopsy as a method to evaluate liver fibrosis and MRI liver scans to assess the LFC. The flow of article selection is shown in *Figure 1*.

The meta-analysis was conducted on a pooled individual population of 1,552 from 13 studies: 1,001 individuals received dual GIP/GLP-1RA, GCGR/GLP-1RA or GLP-1RA and 551 received placebo or active control. Two studies (Loomba et al. and Gastadelly et al.) used dual GIP/GLP-1RA, and two studies (Sanyal et al. and Harrison et al.) used dual glucagon/GLP-1RA as interventions.^20,22–24^ The remaining studies used single GLP-1RA. Among the thirteen studies, six (Loomba et al., Armstrong et al., Sanyal et al., Newsome et al., Bizino et al. and Harrison et al.) used placebo as the control arm; three studies (Gastadelly et al., Jinhua et al. and Liu et al.) used insulin as the control arm; and five studies (Dutour et al., Feng et al., Jinhua et al., Kuchay et al. and Sathyanarayana et al.) used oral antihyperglycaemic agents as the control arm.^11,12,20,22–31^ The duration of follow-up ranged from 12 to 52 weeks. The baseline characteristics of the studies included in the analysis are summarized in *Table 1*.^11,12,20,22–31^

### Outcome measures

Five studies reported the reversal of liver fibrosis degree using liver biopsy. All studies showed the superiority of dual GIP/GLP-1RA, GCGR/GLP-1RA or GLP-1RA to reverse the liver fibrosis degree by at least one stage in individuals with MASLD and T2D. The total odds ratio (OR) with a random effects model (OR 3.72; 95% CI: 2.72, 5.09) was statistically significant (p<0.001). All studies have a low risk of bias, except for Shao et al.^9^ The forest plot analysis and risk of bias are shown in *Figure 2*.^1,12,20,22,23,29,32,33^

Eleven studies reported changes in LFC using MRI scans. All studies showed the superiority of dual GIP/GLP-1RA, GCGR/GLP-1RA or GLP-1RA in decreasing the intrahepatic lipid content in individuals with MASLD and T2D. The MD in the percentage change from baseline (MD -18.90; 95% CI: -18.43, -19.37) was statistically significant (p<0.001). Overall, the studies had a low risk of bias. The forest plot analysis and risk of bias are shown in *Figure 3*.^11,19,20,22–28,30–33^

The subgroup analysis showed that dual GIP/GLP-1RA (OR 28.90; 95% CI: 27.97, 29.83) and GCGR/GLP-1RA (OR 35.31; 95% CI: 34.26, 36.36) have greater efficacy in LFC reduction compared with single GLP-1RA (OR 8.23; 95% CI: 7.60, 8.87). The forest plot analysis is shown in *Figure 4*.^11,19,20,22–28,30–33^

## Discussion

Currently, there is no US Food and Drug Administration-approved treatment for MASLD. Only 3–6% individuals achieved weight loss with lifestyle modification, and most fail to maintain a healthy lifestyle.^8,34^ The pathological hallmark of MASLD includes insulin resistance, obesity and increased fat content. GLP-1RA-based agents represent a potential strategy for treating both MASLD and T2D.^35^ GLP-1RAs lead to a reduction in leptin, resistin and monocyte chemoattractant protein-1 and increase adiponectin levels, which inhibit lipolysis and reduce fat mass. This mechanism leads to a reduction in gluconeogenesis and triglyceride synthesis.^22,36^ GIP and GCGRs also induce lipoprotein lipase to eliminate triglyceride chylomicrons. The reduced incretin effect results in further damage to hepatocytes through increased hepatic insulin resistance, *de novo* lipogenesis and hepatic fat deposition.^36,37^

Treatment with dual GIP/GLP-1RA, GCGR/GLP-1RA and GLP-1RA for MASLD shows good effectiveness in reducing liver fat accumulation and alleviating steatohepatitis. This is further augmented by reductions in inflammation and apoptosis, promoting improved tissue remodelling in the liver.^3,22^ A recently conducted meta-analysis has revealed an improvement in liver fibrosis and intrahepatic lipid accumulation. The meta-analysis by Mantovani et al. reported greater histological resolution of NASH without worsening liver fibrosis (pooled OR 4.06; 95% CI: 2.52, 6.55) with GLP-1RA.^38^ These findings are consistent with some previous studies that reported tirzepatide effectiveness in improving MASLD biomarkers in patients with T2D.^32,39^ Okuma et al. reported that tirzepatide caused significant reduction in HbA1c levels, fatty liver index and fibrosis (FIB-4) index after 6 months of therapy in individuals with MASLD and T2D.^32^

Our meta-analysis shows the superiority of dual GIP/GLP-1RA and GCGR/GLP-1RA to reduce LFC content compared with single GLP-1RA. This is because the activation of GIP receptors in subcutaneous adipose tissue improves insulin sensitivity by increasing postprandial triglyceride uptake and reducing ectopic fat deposition in the liver. Tirzepatide may have direct protective effects on various tissues that play a role in MASLD pathology.^40,41^ Furthermore, glucagon and GIP have direct effects on hepatic fatty acid β-oxidation and lipogenesis, contributing to decreased LFC. Studies suggest that a 30% reduction in LFC is correlated with >2-point reduction in NAFLD activity scores and a 50% LFC reduction is correlated with MASLD resolution (absence of ballooning with minimal to no lobular inflammation) and improved fibrosis.^33,42^ Studies show that survodutide and pemvidutide (dual GCGR/GLP-1RA) are more potent than GLP-1RA because of the appetite suppressing effects of GLP-1RA and the hepatic fat content reduction by GCGR agonism in hepatocyte to synergically enhance liver function and improve fibrosis.^41,43^

To the best of our knowledge, this is the first meta-analysis of dual GIP/GLP-1RA and GCGR/GLP-1RA for MASLD exclusively, conducted on a pooled individual population of 1,552 individuals. As expected, dual GIP/GLP-1RA, GCGR/GLP-1RA and GLP-1RA treatments significantly reversed liver fibrosis and reduced intrahepatic lipid percentage.

This meta-analysis has certain limitations. First, data were analysed from the published effect sizes rather than individual-level pooled data. Second, we only found four RCT articles that studied dual GIP/GLP-1RA and GCGR/GLP-1RA for MASLD. Larger studies are needed to confirm the findings of our study. Third, there is a significant heterogeneity among the included studies. While the major strength of this study is the large number of individuals, it included all treatment modalities for MASLD with T2D as comparison, which are known to have a positive impact on hepatic outcome, and the inclusion of all RCTs to date. We only analysed studies with liver fibrosis analysis by liver biopsy and LFC assessment by MRI liver scan, which can robustly support the clinical implication.

## Conclusion

This meta-analysis suggests that medications combining GIP/GLP-1RA, GCGR/GLP-1RA or using GLP-1RA alone could be beneficial for individuals with both T2D and MASLD. Dual GIP/GLP-1RA and GCGR/GLP-1RA showed superior efficacy in reducing LFC contents compared with single GLP-1RA. These treatments demonstrated a notable reduction in liver fibrosis and fat accumulation within the liver. Although more extensive clinical trials with liver biopsies are needed for confirmation, this analysis provides initial evidence that could influence treatment recommendations for MASLD.
